# Supplementary figures and images for: Cryptic sex in Leishmania depends on SPO11 paralogs
Source: PLoS Pathog. 2026 Apr 29;22(4):e1014181. doi: 10.1371/journal.ppat.1014181 (PMC13143177; doi:10.1371/journal.ppat.1014181)

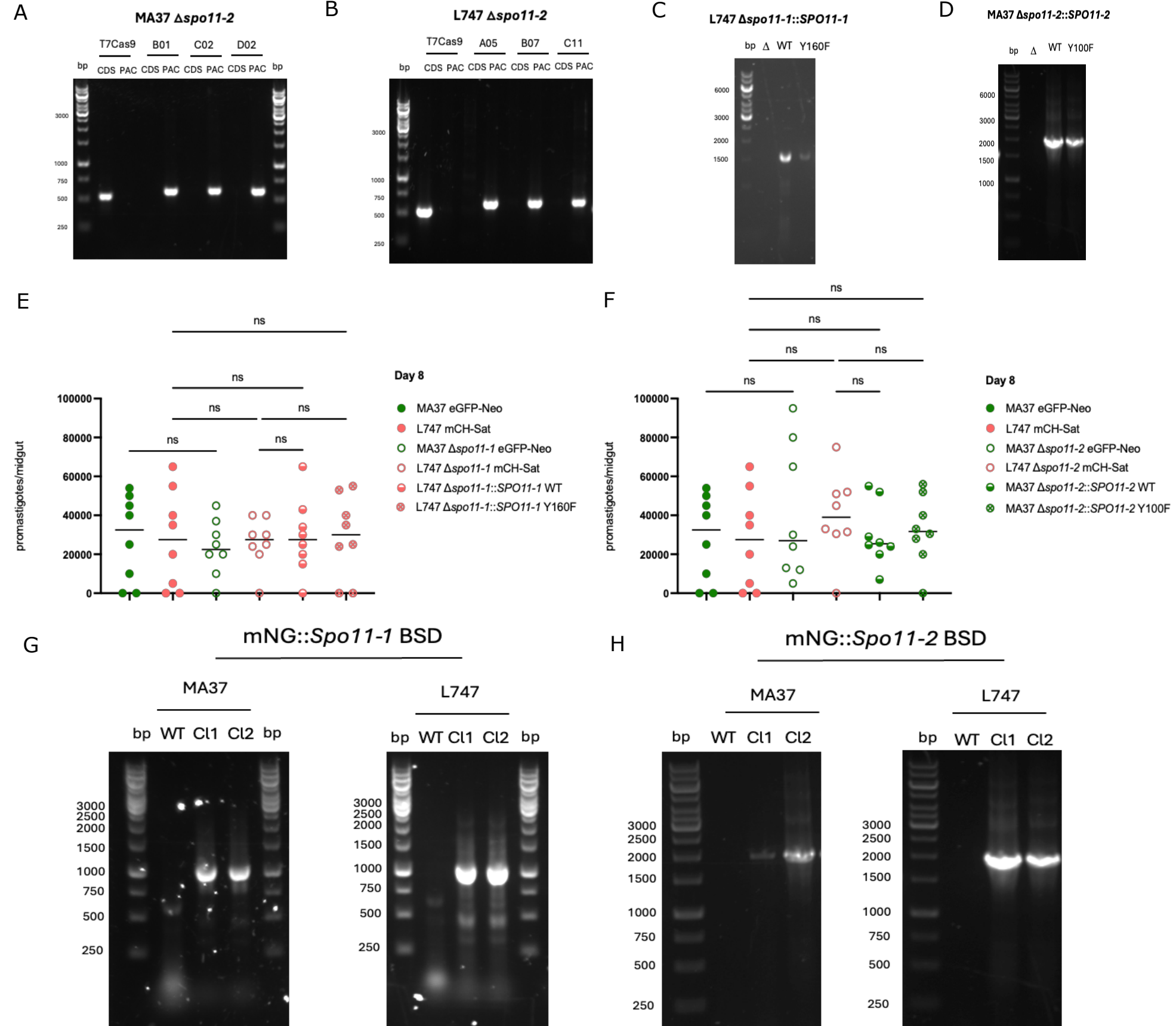

Supplement: S1 Fig — (A–B) PCR confirmation of SPO11–2 deletion in MA37 (A) and L747 (B) clones using CDS-, UTR- and PAC-specific primers. (C–D) PCR genotyping of L747 Δspo11–1::SPO11–1-WT and Δspo11–1::SPO11–1-Y160F (C), and MA37 Δspo11–2::SPO11–2-WT and Δspo11–2::SPO11–2-Y100F (D), confirming integration into the SSU loci. (E–F) Total number of promastigotes per midgut at day 8 post-infection in flies infected with SPO11–1 lines (E) or SPO11–2 lines (F), including parental controls, null mutants, and complemented lines. Each point represents one midgut; horizontal bars show median values. “ns” indicates non-significant comparisons. (G–H) PCR validation of mNeonGreen tagging of SPO11–1 (G) and SPO11–2 (H) in MA37 and L747 clones. WT = wild type; Cl1 and Cl2 = independent tagged clones. (PDF) [file ppat.1014181.s001.pdf]

A

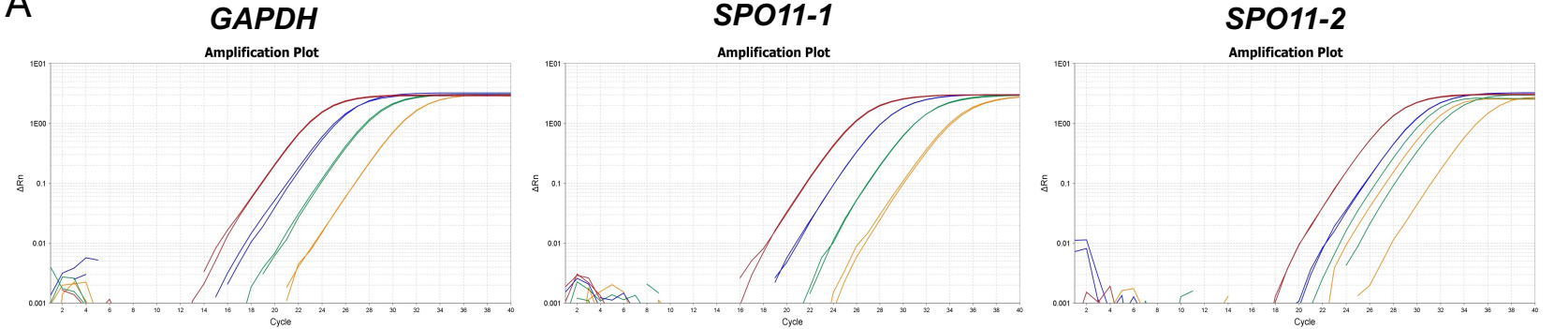

B

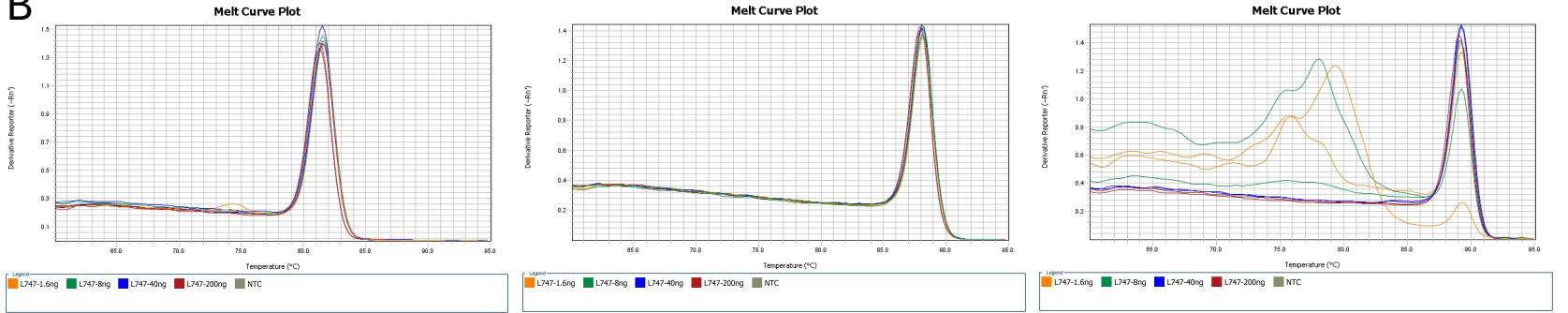

C

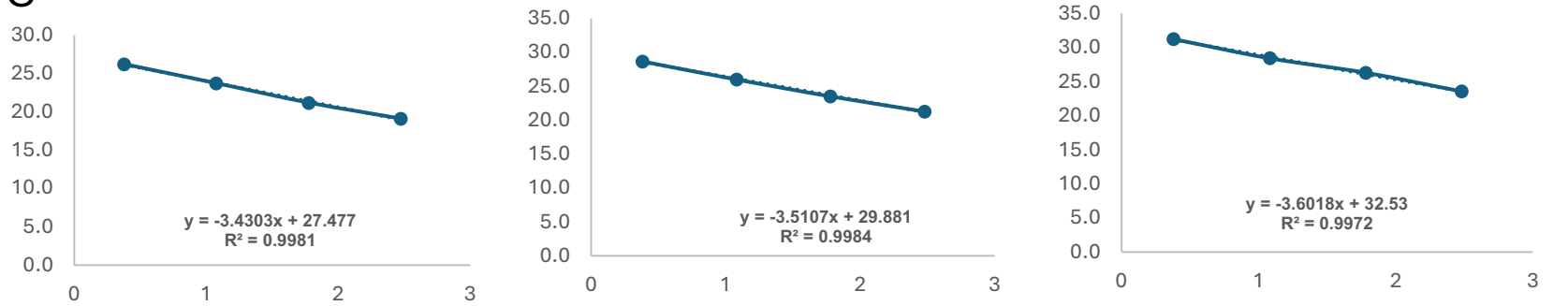

D

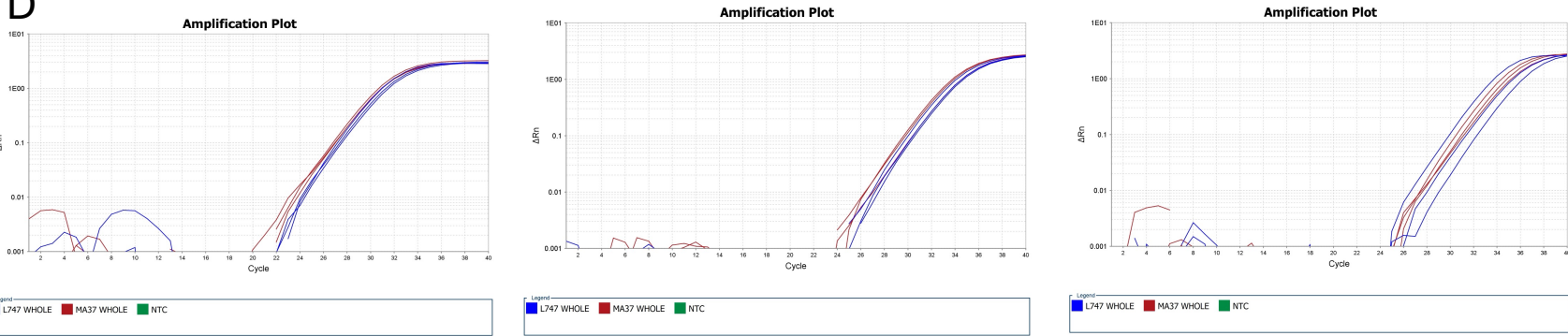

E

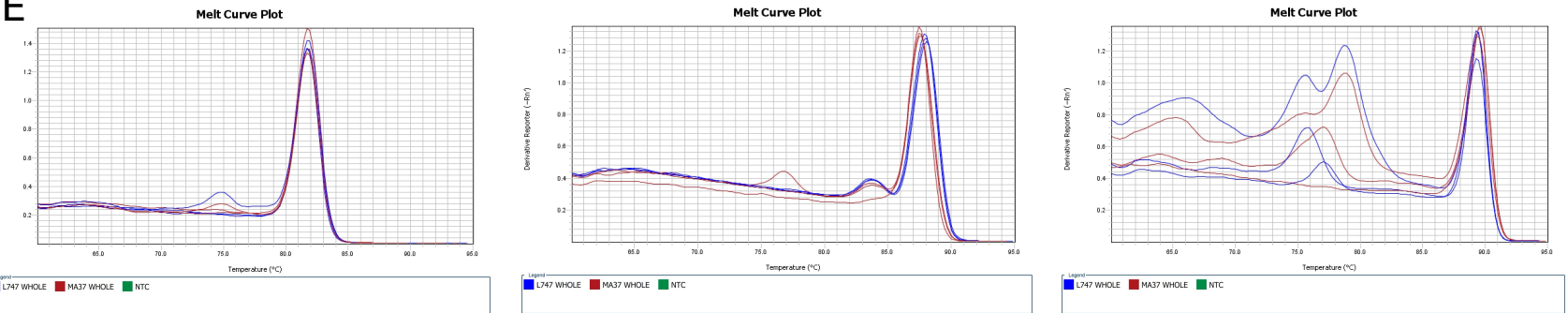

F

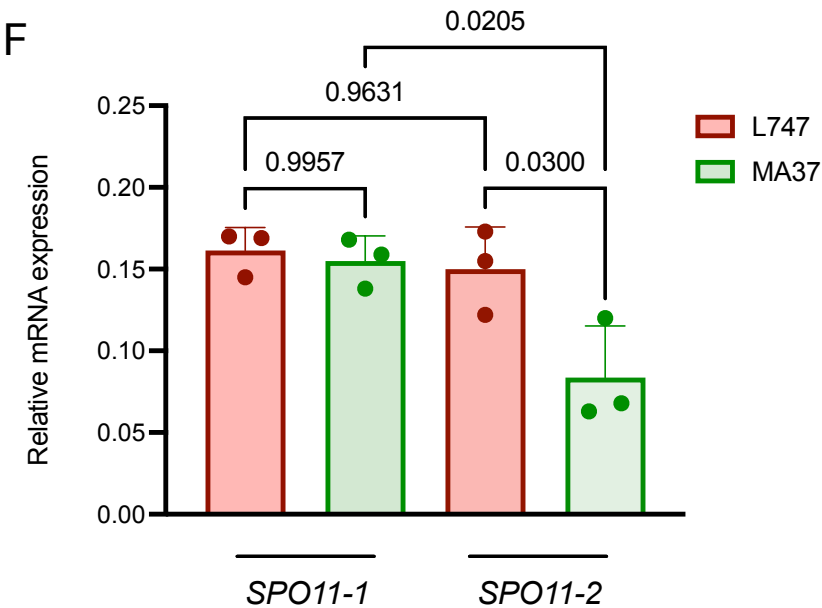

G

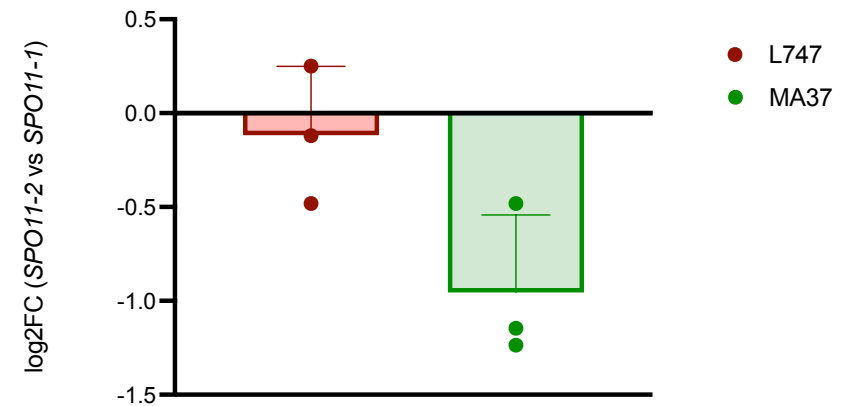

Supplement: S2 Fig — (A) Primer amplification efficiency determined from standard curves generated by five-fold serial dilutions of total RNA for GAPDH (endogenous control), SPO11–1, and SPO11–2. (B) Representative melting curves for GAPDH, SPO11–1, and SPO11–2, demonstrating single, specific amplification products. (C) Standard curves generated from five-fold serial dilutions of total L. tropica RNA and used to calculate primer amplification efficiency for GAPDH (endogenous control), SPO11–1, and SPO11–2. Linear regression lines, slopes, and coefficients of determination (R²) are shown. (D) Amplification plots for experimental samples. (E) Corresponding melting curves for GAPDH, SPO11–1, and SPO11–2 from experimental RT–qPCR reactions performed on infected whole flies. (F) Relative mRNA expression levels of SPO11–1 and SPO11–2 in pools of 40 Lu. longipalpis infected with either the MA37 or L747 L. tropica strain, measured by RT–qPCR. Bars represent the mean of three independent biological replicates, with individual data points overlaid; values were normalized to GAPDH. (G) log₂ fold change in SPO11–1 and SPO11–2 expression in L747 relative to MA37, calculated from the RT–qPCR data shown in panel G. Values represent means from three independent experiments. (PDF) [file ppat.1014181.s002.pdf]

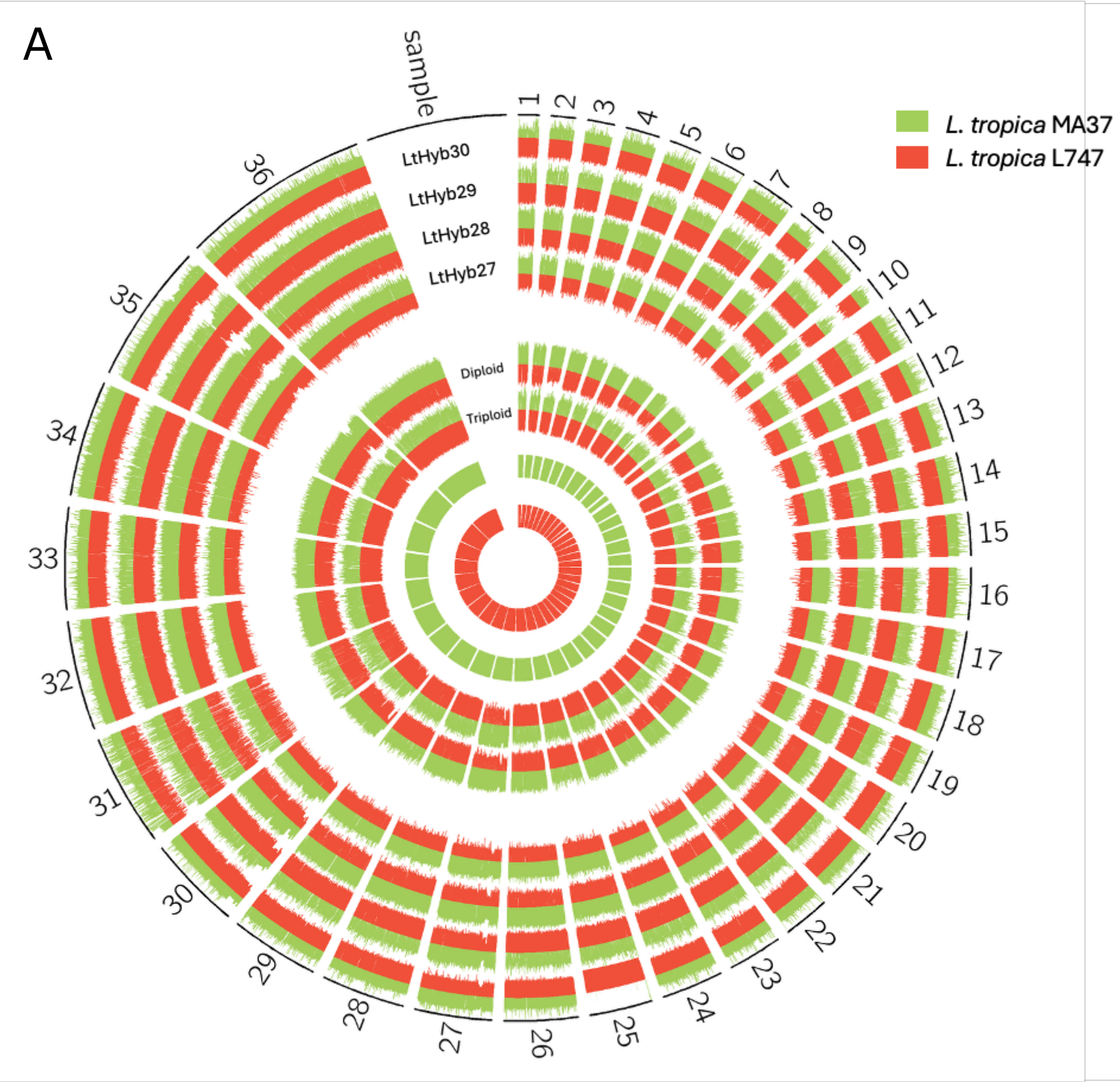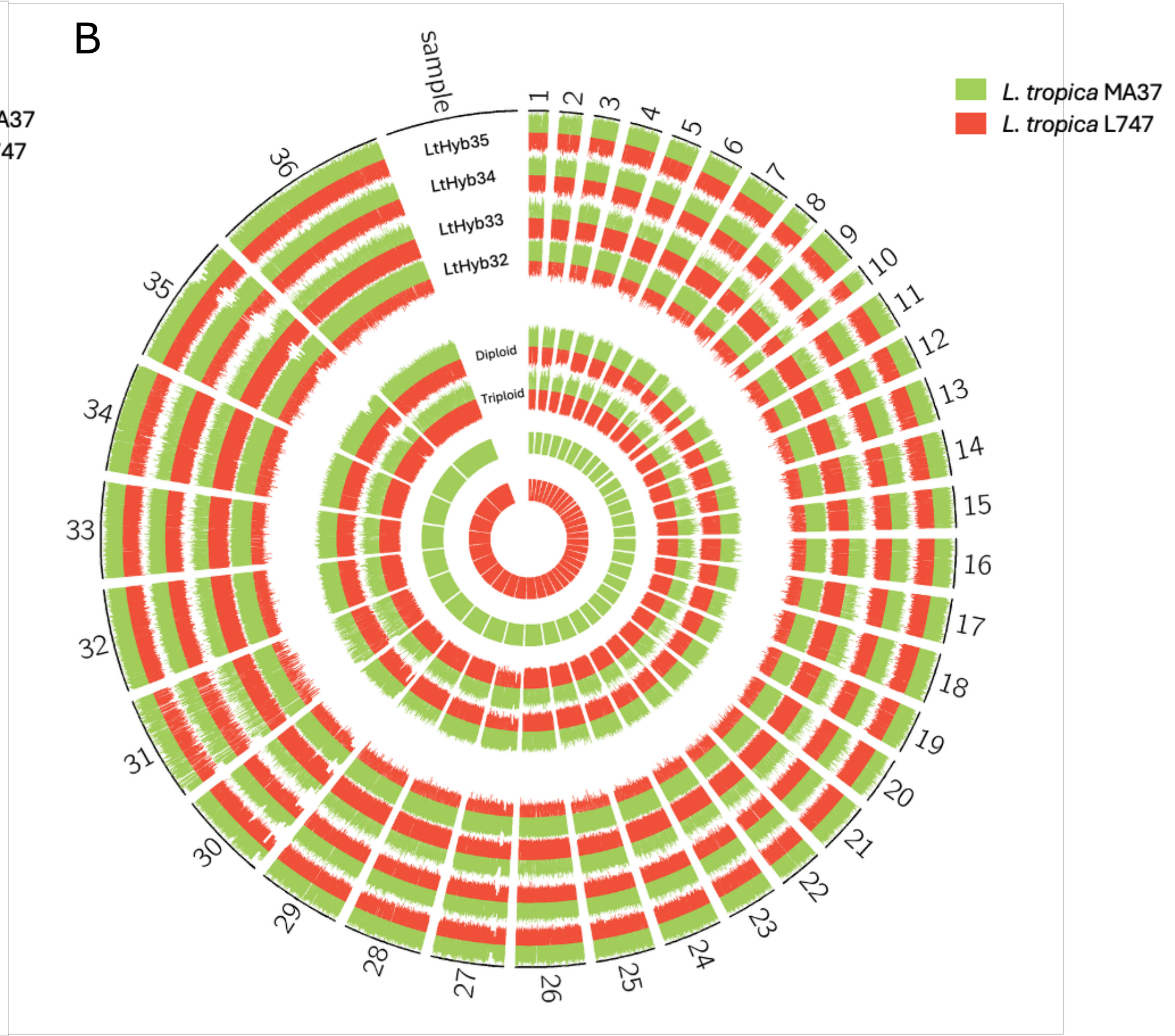

Supplement: S3 Fig — Representative flow cytometry histograms of propidium iodide (PI)–stained parasites illustrating how hybrid DNA content was classified. Diploid, triploid, and tetraploid control lines were used to define reference peaks corresponding to 2n, 3n, and 4n DNA content, respectively. Hybrid populations were classified by comparison to these controls. Multiple representative profiles are shown for triploid hybrids, illustrating the range of DNA content distributions observed among independently derived hybrid clones recovered from SPO11-deficient crosses. (PDF) [file ppat.1014181.s003.pdf]
