# Supplementary material for: Cryptic sex in Leishmania depends on SPO11 paralogs
Source: PLoS Pathog. 2026 Apr 29;22(4):e1014181. doi: 10.1371/journal.ppat.1014181 (PMC13143177; doi:10.1371/journal.ppat.1014181)
Supplement: Attachment — Submitted filename: Response to Reviewers_Catta-Preta et al. 2026_PlosPathogens.docx [file ppat.1014181.s009.docx]

**Additional Editor Comments:**
As you will see from the reports, the reviewers are enthusiastic about the work. We invite you to submit a revised version of the manuscript addressing the minor comments raised.

**R:** We thank you and the reviewers for the thoughtful and constructive evaluation of our manuscript. We are pleased that the reviewers found the study to be methodologically robust, clearly presented, and a valuable contribution to understanding the mechanisms of sexual reproduction in *Leishmania*. We have carefully revised the manuscript to address all comments raised, and we believe these revisions have strengthened the clarity and interpretation of our findings. Below, we provide a detailed, point-by-point response to each comment, along with a summary of the changes implemented in the revised version.

**Journal Requirements:**

1) Please ensure that the CRediT author contributions listed for every co-author are completed accurately and in full.
At this stage, the following Authors/Authors require contributions: Carolina Moura Costa Catta-Preta, Vitor Luiz Da Silva, Claudio Meneses, Kashinath Ghosh, and David L. Sacks. Please ensure that the full contributions of each author are acknowledged in the "Add/Edit/Remove Authors" section of our submission form.
The list of CRediT author contributions may be found here: <https://journals.plos.org/plospathogens/s/authorship#loc-author-contributions>

**R:** We have now completed and verified the CRediT author contributions for all authors in the submission system.

The contributions are as follows:

**Conceptualization:** Carolina Moura Costa Catta-Preta, David L. Sacks
 **Methodology:** Carolina Moura Costa Catta-Preta
 **Investigation:** Carolina Moura Costa Catta-Preta, Vitor Luiz Da Silva
 **Formal Analysis:** Carolina Moura Costa Catta-Preta, Vitor Luiz Da Silva
 **Resources:** Carolina Moura Costa Catta-Preta, Kashinath Ghosh, Claudio Meneses, David L. Sacks
 **Writing – Original Draft:** Carolina Moura Costa Catta-Preta
 **Writing – Review & Editing:** Carolina Moura Costa Catta-Preta, David L. Sacks
 **Supervision:** David L. Sacks
 **Funding Acquisition:** David L. Sacks

2) Please provide an Author Summary. This should appear in your manuscript between the Abstract (if applicable) and the Introduction, and should be 150-200 words long. The aim should be to make your findings accessible to a wide audience that includes both scientists and non-scientists. Sample summaries can be found on our website under Submission Guidelines:
<https://journals.plos.org/plospathogens/s/submission-guidelines#loc-parts-of-a-submission>

**R:** An Author Summary has already been included in the manuscript, in accordance with the journal guidelines (150–200 words, placed between the Abstract and the Introduction). We have reviewed the section to ensure that it clearly communicates the main findings to a broad audience, including both scientists and non-specialists.

3) We do not publish any copyright or trademark symbols that usually accompany proprietary names, eg ©,  ®, or TM  (e.g. next to drug or reagent names). Therefore please remove all instances of trademark/copyright symbols throughout the text, including:
- ® on page: 12 and 15.

**R:** All instances of trademark/copyright symbols (®) have now been removed from the manuscript, including those on pages 12 and 15.

4) Please upload all main figures as separate Figure files in .tif or .eps format. For more information about how to convert and format your figure files please see our guidelines: 
<https://journals.plos.org/plospathogens/s/figures>

**R:** All main figures have now been uploaded as separate files in the required .tiff format, in accordance with the journal guidelines

5) Please amend your detailed Financial Disclosure statement. This is published with the article. It must therefore be completed in full sentences and contain the exact wording you wish to be published.
- State the initials, alongside each funding source, of each author to receive each grant. For example: "This work was supported by the National Institutes of Health (####### to AM; ###### to CJ) and the National Science Foundation (###### to AM)."
- State what role the funders took in the study. If the funders had no role in your study, please state: "The funders had no role in study design, data collection and analysis, decision to publish, or preparation of the manuscript.".
If you did not receive any funding for this study, please simply state: u201cThe authors received no specific funding for this work.u201d

**R:** The Financial Disclosure statement has now been revised as follows:

This work was supported in part by the Intramural Research Program of the National Institute of Allergy and Infectious Diseases, National Institutes of Health (to D.L.S.). The funders had no role in study design, data collection and analysis, decision to publish, or preparation of the manuscript.

**Reviewers' Comments:**

Reviewer's Responses to Questions

**Part I - Summary**
Please use this section to discuss strengths/weaknesses of study, novelty/significance, general execution and scholarship.

Reviewer #1: Very little is known about the mechanism of meiosis in kinetoplastids, which are evolutionarily divergent from commonly studied model eukaryotes such as yeast and humans. For example, in Leishmania, formation of a gamete with a haploid genome has not been observed. This manuscript by Catta-Preta and colleagues reports characterization of two paralogs of a key meiotic recombination protein SPO11 (SPO11-1 and SPO11-2) for hybridization in Leishmania tropica (using L747 and MA37 parental strains that allow SNP analysis). By analyzing hybridization frequencies and performing whole genome sequencing of hybrids that derived from the parents whose SPO11-1 or SPO11-2 genes are deleted, they demonstrate the importance of the catalytic activity of these SPO11 proteins. Importantly, their somy analysis showed a genome-wide skew toward the SPO11-deficient parent, which could be interpreted as an indirect evidence for SPO11-dependent haploidization in Leishmania.
Overall the manuscript is well written and structures well presented. A previous paper by these authors published in Nature communications (2023) used similar techniques to demonstrate the importance for other key meiotic factors (HOP1 and HAP2) in hybridization. In this sense, one may feel that the progress reported in the current manuscript is rather incremental. However, given how little is known about the meiotic mechanism in Leishmania and kinetoplastids in general, I would like to strongly support the publication of this manuscript in Plos Pathogens. Below are minor comments, which the authors may consider to address in the revised manuscript.

- Are the SPO11-1 and SPO11-2 protein and mRNA (coding and UTRs) sequences identical in the L747 and MA37 lines?
- Line 183, perinuclear compartment rather than in the nucleus: Do the authors know that the mNG-tagged proteins are non-functional? I have an impression that this localization pattern is actually similar to what the authors observed for HOP1 in their 2023 paper (Figure 6). Could the obtained images represent a genuine localization of SPO11 proteins?

Bungo Akiyoshi

**R:** We thank the reviewer for the careful evaluation of our manuscript and for the positive and thoughtful comments. We appreciate the recognition of the relevance of this work in advancing our understanding of meiosis-related processes in *Leishmania*, a system in which these mechanisms remain poorly understood.

**1. Are the SPO11-1 and SPO11-2 protein and mRNA (coding and UTRs) sequences identical in the L747 and MA37 lines?**

We thank the reviewer for this important question. The *SPO11-1* and *SPO11-2* coding sequences are highly conserved between L747 and MA37, but not identical. We identified a limited number of amino acid substitutions between the two strains, with no obvious disruption of conserved domains.

We have clarified this point in the revised manuscript. We also note that variations in non-coding regions (including UTRs) may further contribute to potential differences in expression or regulation between strains. Please see lines 431-433.

**2. Line 183, perinuclear compartment rather than in the nucleus: Do the authors know that the mNG-tagged proteins are non-functional? Could the obtained images represent a genuine localization of SPO11 proteins?**

We thank the reviewer for this insightful comment. We agree that the observed localization pattern is similar to what we previously reported for HOP1 (Catta-Preta et al., 2023), and it is therefore possible that this represents a biologically relevant localization.

However, we note that the mNeonGreen-tagged SPO11 lines did not rescue the hybridization phenotype in the corresponding knockout background, suggesting that the tagged proteins are not fully functional. For this reason, we interpreted the observed localization with caution in the manuscript. We have now clarified this point in the revised text and explicitly acknowledge the possibility that the perinuclear signal may still reflect, at least in part, the native localization of SPO11 proteins, while also noting the limitations associated with the tagged constructs.

**R:** We agree that the observed localization pattern is reminiscent of what we previously reported for HOP1 (Catta-Preta et al., 2023), and it is therefore possible that this represents a biologically relevant localization.

At present, we do not have functional data to determine whether the mNeonGreen-tagged SPO11 proteins fully retain their native activity. For this reason, we have interpreted the localization results with caution in the manuscript. We have now clarified this point in the revised text and explicitly acknowledge that the observed perinuclear signal may reflect the native localization of SPO11 proteins (lines 189-191).

Reviewer #2: This is an interesting paper. The existence of sex in Leishmania has been known, or inferred, for many years. However, the mechanisms involved are only slowly being dissected. The role of the process in generating parasite diversity has possible implications stretching from disease pathogenesis to drug resistance. This paper focuses on the role of the meiotic endonuclease SPO11, a widely conserved protein which has a central role in generating the double stranded DNA breaks that are required to initiate recombination. Overall, the paper is well written, the experiments are competently performed, and the conclusions are supported by the data. In preparing a final version of the manuscript, there are a few points which the authors should clarify or comment on, as set out below.

**R:** We thank the reviewer for the thoughtful evaluation of our manuscript and for the positive assessment of the study. We appreciate the recognition of the importance of understanding the mechanisms of sexual reproduction in *Leishmania* and the role of SPO11 in this process.

We have carefully considered the reviewer’s comments and have addressed each point to improve the manuscript.

Reviewer #3: The authors have made key contributions to our understanding of cryptic sex in Leishmania and in the presented study they provide a clear and rigorous demonstration of the importance of the conserved meiotic endonuclease SPO11, present in this parasite as two paralogs, SPO11-1 and SPO11-2. The roles of these paralogs are investigated in in vivo sandfly infections using a well-integrated experimental strategy that includes gene expression analysis with tagged versions of the proteins, gene knockouts with complementation by wild-type and catalytically inactive variants, and genome-wide analysis of hybrid progeny. The study is methodologically robust and logically coherent. The data convincingly establish that SPO11-dependent DNA double-strand break formation is central to Leishmania hybridization, while also revealing strain-specific functional differences between the paralogs.
Given that the sexual cycle in the vector remains cryptic, without direct observation of hybridization, haploid gametes, or defined meiotic stages, and that alternative mechanisms such as parasexuality have been proposed, this work provides important mechanistic insight. Building on prior identification of factors involved in chromosome pairing and hybrid formation, the authors demonstrate that both SPO11 paralogs are required in each mating partner for efficient hybridization. Loss of either paralog leads to aberrant hybrids with unbalanced parental contributions and widespread aneuploidy.

**R:** We are grateful to the reviewer for the detailed and generous evaluation of our work. We particularly appreciate the recognition of the experimental approach and the interpretation of SPO11 function in the context of *Leishmania* hybridization.

We address the specific points raised below and have revised the manuscript accordingly to improve clarity and precision.

**Part II – Major Issues: Key Experiments Required for Acceptance**
Please use this section to detail the key new experiments or modifications of existing experiments that should be absolutely required to validate study conclusions.

Generally, there should be no more than 3 such required experiments or major modifications for a "Major Revision" recommendation. If more than 3 experiments are necessary to validate the study conclusions, then you are encouraged to recommend "Reject".

Reviewer #1: (No Response)

Reviewer #2: NA

Reviewer #3: The study is methodologically and conceptually robust, with no major issues identified.

**Part III – Minor Issues: Editorial and Data Presentation Modifications**
Please use this section for editorial suggestions as well as relatively minor modifications of existing data that would enhance clarity.

Reviewer #1: (No Response)

Reviewer #2: 1. In Fig. 1, which describes the extent of hybrid formation, the violin plots in (A) and (B) of the control cross (MA37 T7Cas9 eGFP-Neo + L747 T7Cas9 mCH-Sat) display a different profile. Are these differences significant?

**R:** The apparent differences in the distribution profiles between panels (A) and (B) reflect variability between independent experiments rather than a consistent or statistically significant difference between conditions. Statistical analysis did not reveal a significant difference in hybridization frequency for the control cross across these datasets.

2. The manuscript reports that N-terminal tagging of SPO11-1 and SPO11-2 apparently disrupts nuclear targeting of the proteins, but concludes that the pattern of expression provides a reliable temporal readout of the process. This is probably a reasonable assumption, but the authors should mention the caveat that they cannot exclude the possibility that mis-targeting may have an impact on the extent of protein turnover. It would also be interesting to hear the author’s views on why only a small percentage of the parasites express detectable protein, and what the mechanism(s) for this might be. This could be mentioned at the appropriate place in the Results, or in the Discussion.

**R:** We agree that N-terminal tagging may influence not only protein localization but also protein stability and turnover. We have now added a statement in the Results to explicitly acknowledge that we cannot exclude potential effects of mis-targeting on protein turnover (lines 194-195).

Regarding the observation that only a small fraction of parasites express detectable protein, we propose that SPO11 expression may be restricted to a transient and specialized subpopulation undergoing sexual differentiation, consistent with the temporally limited window of hybrid formation in the sand fly.

3. The differences in DNA content of the hybrid populations is intriguing. Have the authors checked on the infectivity of these parasite lines, and do they know if there is an impact on genome content as a result of continuous rounds of macrophage infection in vitro, or even as a result of experimental mouse infections. It would be nice to see some information on infectivity, but it is probably unreasonable to insist on genome stability data, if they are not available.

**R:** We thank the reviewer for this interesting point. We agree that the differences in DNA content raise important questions regarding the biological properties and stability of these hybrid populations.

At present, we have not assessed the infectivity of these hybrid lines in macrophage or mouse infection models. Similarly, we have not evaluated genome stability following serial passage in vitro or in vivo. However, previous work from our group has shown that *Leishmania* hybrids maintain stable genome content, with no loss of ploidy, following sand flies and mouse infection (Louradour et al., 2020, Cell Reports). These observations suggest that the altered ploidy states observed here are likely to be stably maintained, although this was not directly tested for the hybrids generated in this study.

Reviewer #3: Introduction:

Page 2 – lines 61- 63. For this sentence, it would be helpful to provide a reference to allow readers to easily access the article.

**R:** We have now added an appropriate reference to support this statement.

Page 3 – line 83 – “The transmission cycle of leishmanial diseases can be anthroponotic or zoonotic”. While this is correct, the statement may be misleading, as leishmaniasis is predominantly zoonotic, with anthroponotic transmission limited to specific geographic regions and parasite species. I suggest clarifying or rephrasing this sentence to better reflect this distinction.

**R:** We have revised the sentence to more accurately reflect the predominance of zoonotic transmission, while noting that anthroponotic transmission occurs in specific geographic regions and parasite species (lines 85-87).

Page 3 – lines 85-86: “The different clinical presentations have particular Leishmania strain and species associations, with over 20 species pathogenic for humans.” Here again, the issue is one of emphasis. I recommend focusing on species rather than strains, as species-level differences are the primary determinants of clinical manifestations. Accordingly, “strains” could be omitted to improve clarity and accuracy.

**R:** We thank the reviewer for this helpful suggestion. We agree that species-level differences are major determinants of clinical manifestations in *Leishmania*. However, we have chosen to retain reference to strains, as accumulating evidence indicates that intraspecies variation, including strain differences and hybridization, can also influence disease outcome. We therefore believe that the current phrasing more accurately reflects the biological complexity of the system.

Page 6, second paragraph (lines 197–205): there appear to be two incorrect figure references. Where the text cites Figure S3, it likely should be Figure S2; similarly, the reference to Figure 3A also appears to be incorrect and should be revised accordingly.

**R:** We thank the reviewer for catching these errors. We have corrected the figure citations in this section of the revised manuscript.
